# Evaluation and subgroup analysis of the efficacy and safety of intensive rosuvastatin therapy combined with dual antiplatelet therapy in patients with acute ischemic stroke

**DOI:** 10.1007/s00228-022-03442-8

**Published:** 2022-12-29

**Authors:** Ting Deng, Tong Zhang, Haitao Lu, Jingmian Chen, Xiaomeng Liu, Wei He, Xiaohua Yao

**Affiliations:** 1 Emergency Department, China Rehabilitation Research Center Beijing Bo’ai Hospital, Beijing, 100068 China; 2 Neurology Department, China Rehabilitation Research Center Beijing Bo’ai Hospital, Beijing, 100068 China

**Keywords:** Intensive rosuvastatin, Dual antiplatelet therapy, Recurrent ischemic stroke, Subgroup analysis

## Abstract

**Objectives:**

We investigated the efficacy of intensive rosuvastatin therapy plus 7-day dual antiplatelet therapy (DAPT) in reducing stroke recurrence for patients with acute ischemic stroke (AIS) and compared subgroups of patients.

**Methods:**

We enrolled patients with AIS whose time of onset to medication was ≤ 72 h, and the baseline scores of NIHSS (bNIHSS) were 0–10. The patients received intensive rosuvastatin therapy plus 7-day DAPT with aspirin and clopidogrel (study group) or rosuvastatin plus single antiplatelet therapy (SAPT, control group). The primary outcomes were recurrence of ischemic stroke, bleeding, statin-induced liver injury, and statin-associated myopathy (SAM) within 90 days. We also performed a subgroup analysis to assess the heterogeneity of the two therapy regimens in reducing recurrent stroke.

**Results:**

Recurrent stroke occurred in 10 patients in the study group and 42 patients in the control group (hazard ratio [HR], 0.373, 95% confidence interval [CI], 0.178–0.780; *P* = 0.009). Bleeding events occurred in 9 patients in the study group and 14 patients in the control group (HR, 1.019; 95%CI, 0.441–2.353; *P* = 0.966). Statin-induced liver injury and SAM were not recorded. Intensive rosuvastatin plus 7-day DAPT was generally effective in reducing the risk of recurrent stroke, except in the subgroup with bNIHSS ≤ 2. The therapy was particularly efficient in the elderly, male, high-bNIHSS, and hypertension, diabetes, and hyperlipidemia subgroups, with *P* < 0.02.

**Conclusions:**

Without increasing bleeding and statin-associated adverse events, intensive rosuvastatin therapy plus 7-day DAPT significantly reduced the risk of recurrent stroke, especially for subgroups with high-risk factors. *Clinical trial registration*. China Clinical Trial Registration Center (ChiCTR1800017809).

**Supplementary Information:**

The online version contains supplementary material available at 10.1007/s00228-022-03442-8.

## Introduction

Disorder of lipid metabolism, dysfunction of endothelial cells, and aggregation of many inflammatory factors are the external conditions of arterial thrombosis [1, 2], while platelet activation is the internal factor [3]. All these factors interact with and promote each other [4], eventually leading to acute coronary syndrome (ACS) and acute ischemic stroke (AIS).

Intensive statin therapy is commonly combined with dual antiplatelet therapy (DAPT) in patients with ACS after percutaneous coronary intervention, stent implantation [5, 6], and AIS [7, 8]. This combination treatment can significantly reduce the risk of recurrent thrombosis after stent implantation [9] and recurrent ischemic stroke after AIS [10, 11]. However, there are few reports on the efficacy of this regimen among different subgroups of patients with AIS. Thus, we investigated the risks and benefits of intensive rosuvastatin therapy plus 7-day DAPT in reducing recurrent ischemic stroke for patients with mild to moderate AIS and compared subgroups.

## Materials and methods

### Patients

We recruited patients aged 18 years or older who were admitted to the Emergency Department of our hospital from October 2016 to December 2019. Computed tomography (CT) and magnetic resonance imaging (MRI) of the head was used to confirm the new focal infarction lesions within 72 h after the onset. Their National Institutes of Health Stroke Scale (NIHSS) scores at registration were 0–10. We excluded patients with intravenous thrombolysis/arterial thrombectomy or anticoagulation treatments, patients undergoing menstruation or pregnancy, and patients preparing for pregnancy within 3 months. In total, 331 patients with AIS met the above criteria, including 204 patients in the control group and 127 patients in the study group according to the therapy regimens. The Medical Ethics Committee of the China Rehabilitation Research Center approved this study (Ethics approval number: 2018–022-1). All patients provided written informed consent.

### Study design and treatment

We stratified the patients based on their baseline demographic characteristics (Table 1). They received one of the two therapy regimens on a voluntary basis (DAPT + intensive rosuvastatin therapy for the study group and single antiplatelet therapy (SAPT) + rosuvastatin for the control group). The follow-up time was 90 days. The primary outcomes were a new ischemic stroke, bleeding events, and the adverse events statin-induced liver injury or statin-associated myopathy (SAM) within 90 days; the secondary outcome was the heterogeneity of the two therapy regimens in reducing recurrent ischemic stroke among subgroups of patients with AIS.

**Table 1:** Stratifying basis for subgroups

**Indicators**	**Cut-off**	**Assign value**
Age, years^a^	68 years old	≤ 68 years old = 0, > 68 years old = 1
Gender	-	Female = 0, male = 1
OMT, hours^b,d^	48 h	≤ 48 h = 0, > 48 h = 1
SBP at registration^e^	140 mm Hg	≤ 140 mm Hg = 0, > 140 mm Hg = 1
**History of diseases**		
Hypertension	-	No = 0, yes = 1
Diabetes mellitus	-	No = 0, yes = 1
Hypercholesterolemia	-	No = 0, yes = 1
Known atrial fibrillation	-	No = 0, yes = 1
Prior-stroke	-	No = 0, yes = 1
Prior antiplatelet	-	No = 0, yes = 1
bNIHSS^c,f^	2 points	≤ 2 points = 0, > 2 points (3–10 points) = 1

^a^By the end of 2015, the average life expectancy in China reached 76.34 years [12], with an increase of 1 year every 5 years [13]. The average life expectancy in 2016–2019 was 68–69 years. Therefore, the cut-off age was 68 years

^b^The Chinese Stroke Guidelines [14] only recommend DAPT for AIS patients within 48 h of symptom onset. Therefore, the cut-off time of onset to medication (OMT) was 48 h

^c^When the NIHSS changes by less than 2 points, the symptoms change of focal neurological deficit is not obvious [15]. In addition, whether intravenous thrombolysis or not did not affect the prognosis of AIS patients with NIHSS scores ≤ 2 [16], so the cut-off value of the baseline scores of NIHSS at recruitment (bNIHSS) was 2

^d^OMT, time of onset to medication

^e^SBP, systolic blood pressure

^f^bNIHSS, the baseline scores of National Institute of Health stroke scale at recruitment

### Therapy regimens

Patients in the study group received DAPT + intensive rosuvastatin therapy: aspirin (Bayer, 100 mg per tablet) 100 mg/d with an initial dose of 300 mg for 90 days, clopidogrel (Sanofi, 75 mg per tablet) 75 mg/d with an initial dose of 75–300 mg determined based on the clinical symptoms for 7 days, plus rosuvastatin (Nanjing Chia Tai-Tianqing Pharmaceutical Co., Ltd, 10 mg per tablet), 20 mg/d for 21 days, and then 10 mg/d for 90 days in total. Patients in the control group received SAPT + rosuvastatin: aspirin (Bayer, 100 mg per tablet) 100 mg/d or clopidogrel (Sanofi, 75 mg per tablet) 75 mg/d for 90 days, plus rosuvastatin (Nanjing Chia Tai-Tianqing Pharmaceutical Co., Ltd, 10 mg per tablet) 10 mg/d for 90 days.

According to previous studies, aspirin and clopidogrel consistently reduced recurrent vascular events [17] or recurrent ischemic stroke events [18, 19] for stroke patients within 1 year. For a few patients in the control group who were intolerant to aspirin, we used clopidogrel 75 mg/d instead of aspirin 100 mg/d due to its lower gastric toxicity [17], which had no significant effect on the outcome.

### Assessment criteria

We assessed focal neurological deficits by assessing the bNIHSS, which ranges from 0 to 43, with higher scores indicating worse deficits [20]. Because there were few patients with bNIHSS above 10 in our Emergency Department, and the therapy regimens in this study were very poor for them, we only registered patients with bNIHSS ≤ 10 in this study.

Recurrent ischemic stroke—the aggravation of existing clinical symptoms or the emergence of new focal neurological deficit symptoms within 90 days after the first treatment—was confirmed by CT and MRI scans of the head, which showed obviously enlarged original lesions or new ischemic lesions. Bleeding events included intracranial and gastrointestinal mucosal hemorrhage, which were confirmed by head CT and gastric contents analysis or fecal occult blood test, respectively, within 90 days after treatment. According to the global use of streptokinase and tissue plasminogen activator to treat coronary occlusion (GUSTO) [21], the severity of bleeding was classified as mild, moderate, or severe. Statin-induced liver injury and SAM were defined as a more than three-fold increase in the normal upper limit levels of transaminase (alanine transferase [ALT] or aspartate transferase [AST]) [22] and creatine kinase (CK) [23] within 90 days.

### Statistical analysis

This study adopted an incomplete randomized controlled trial design, with Type I Error α = 0.05 and power of test (1 − β) = 0.85. The PASS 15.0 software (NCSS, LLC, Kaysville, UT, USA) was used to estimate the sample size. According to a meta-analysis by Kwok et al. [24], SAPT reduced the recurrence rate of ischemic stroke by 52%. In line with the CHANCE trial [25], DAPT actually reduced AIS recurrent stroke by nearly 84%, which is significantly more than SAPT. With these studies in mind, we concluded that we needed a total sample size of 312 cases, with a control group to study group size ratio of around 3:2. We effectively recruited 331 patients, with 204 patients in the control group and 127 in the research group.

SPSS 25.0 statistical software (IBM Corporation, Armonk, NY, USA) was used for data analysis. We expressed measurement data as the median (M) and inter-quartile range (IQR) from the rank-sum test results and expressed count data as % from the χ^2^ test results. A Cox proportional hazards model was used to evaluate differences in the recurrent ischemic stroke events and bleeding events within 90 days between the two groups. We compared the ALT, AST, lactate dehydrogenase (LDH), and CK levels before and 2 weeks after therapy using a rank sum test. Finally, we assessed the heterogeneity of the two therapy regimens of this study in reducing recurrent ischemic stroke by performing a subgroup analysis. *P* < 0.05 was considered statistically significant for the first three statistical analyses, and *P* < 0.02 was considered statistically significant for the subgroup analysis.

## Results

### Baseline data between the two groups

Baseline demographic characteristics were well balanced between the two groups with all *P* > 0.05 among age; gender; systolic and diastolic blood pressure at registration; OMT; bNIHSS; previous medical histories; and the levels of ALT, AST, LDH, and CK before and 2 weeks (14 ± 3 days) after therapy (Table 2).

**Table 2:** Baseline demographic characteristics of patients

**Characteristic**	**Control (*n* = 204)**	**Study (*n* = 127)**	***P* value**
Median age (IQR), year	67.00 (59.00–82.00)	66.00 (58.50–76.00)	0.117
Female, no. (%)	60 (66.7)	30 (33.3)	0.257
Median SBP (IQR), mm Hg^a^	154.00 (138.00–173.50)	154.00 (143.00–172.00)	0.713
Median DBP (IQR), mm Hg^b^	89.00 (78.00–102.00)	91.00 (81.00–103.00)	0.056
**Medical history, no. (%)**			
hypertension	174 (62.1)	106 (37.9)	0.643
Diabetes mellitus	110 (53.9)	57 (44.9)	0.115
Hyperlipemia	182 (89.2)	106 (83.4)	0.134
Known atrial fibrillation	27 (13.2)	16 (12.6)	1.000
Ischemic stroke	89 (43.6)	44 (34.6)	0.108
Pre-antiplatelet	41 (20.1)	35 (27.6)	0.139
Median OMT (IQR), hour^c^	12.00 (4.00–24.00)	18.00 (4.50–38.00)	0.388
Median bNIHSS (IQR)^d^	3.00 (2.00–4.00)	4.00 (3.00–5.00)	0.076
**Median baseline of various enzymology before medication (IQR), U/L**			
ALT^e^	16.80 (12.05–22.50)	18.40 (13.70–23.70)	0.183
AST^f^	17.05 (12.90–23.10)	16.50 (13.00–21.60)	0.560
LDH^g^	175.50 (157.00–200.50)	179.00 (151.00–202.00)	0.825
CK^h^	81.00 (54.50–120.50)	72.00 (52.00–101.50)	0.099
**Median of various enzymology in 2 weeks after medication(IQR), U/L**			
ALT^e^	16.75 (11.55–24.55)	17.10 (13.00–25.25)	0.466
AST^f^	17.70 (13.30–22.80)	17.60 (14.00–22.55)	0.956
LDH^g^	180.00 (155.50–214.00)	172.00 (151.00–204.00)	0.079
CK^h^	69.50 (49.00–101.50)	68.00 (48.00–101.00)	0.514

^a^SBP, systolic blood pressure

^b^DBP, diastolic blood pressure

^c^OMT, time of onset to medication

^d^bNIHSS, the baseline scores of National Institute of Health stroke scale before medication

^e^ALT, alanine aminotransferase

^f^AST, aspartate aminotransferase

^g^LDH, lactate dehydrogenase

^h^CK, creatine kinase

### Recurrent ischemic stroke

**Fig. 1:**
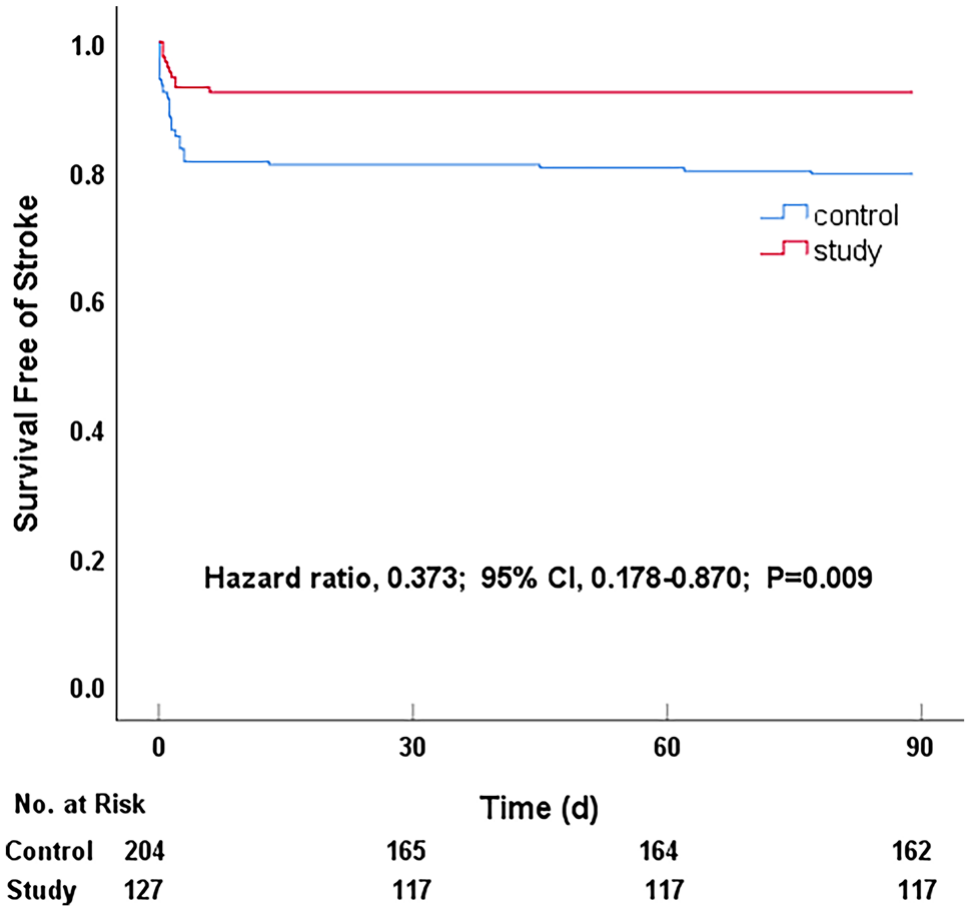
Probability of survival free of recurrent ischemic stroke within 90 days

Within 90 days, 52 patients underwent recurrent ischemic stroke: 10 (7.87%) in the study group and 42 (20.60%) in the control group. The study group had a 62% lower risk of recurrent ischemic stroke than the control group (hazard ratio [HR] for the study group *vs.* control group, 0.373, 95% confidence interval [CI], 0.178–0.780, *P* = 0.009). This result shows that intensive rosuvastatin therapy plus 7-day DAPT was far superior to rosuvastatin plus SAPT in reducing the risk of recurrent ischemic stroke within 90 days (Table 3 and Fig. 1).

**Table 3:** The primary outcome within 90 days

**Outcome**	**Study (*n* = 127)**		**Control (*n* = 204)**		**Hazard ratio** **(95% CI)**	***P* value**
	**Cases with event (no.)**	**Event rate (%)**	**Cases with event (no.)**	**Event rate (%)**		
Stroke	10	7.87	42	20.6	0.373 (0.178–0.780)	0.009
Bleeding^*^	9	7.09	14	6.86	1.019 (0.441–2.353)	0.966

*The bleeding was divided into mild, moderate, and severe bleeding according to GUSTO criteria [21], and all bleeding events were minor bleeding from gastrointestinal mucosa

### Bleeding events

A total of 9 patients (7.09%) in the study group and 14 patients (6.86%) in the control group reported bleeding events. A Cox proportional hazards model revealed no significant difference between the two groups (HR, 1.019; 95% CI, 0.441–2.353; *P* = 0.966), suggesting that intensive rosuvastatin therapy plus 7-day DAPT did not increase the risk of bleeding compared with rosuvastatin plus SAPT (Table 3).

### Statin-induced liver injury or SAM

None of the patients showed an increase superior to three-fold in the levels of ALT, AST, or CK. The ALT, AST, LDH, and CK levels remained stable before therapy and after 2 weeks (14 ± 3 days) among the groups (*P* > 0.05), except for the CK levels in the control group, which were significantly lower after 2 weeks of treatment (*P* < 0.001) (Table 4). These findings showed that the two regimens did not increase the risk of statin-induced liver injury or SAM.

**Table 4:** Comparison of various enzymology before and 2 weeks after medication (comparison of the levels of transaminase and muscle enzymes before and 2 weeks (14 ± 3 days) after treatment intra each group)

		**Control (*n* = 204)**				**Study (*n* = 127)**			
		**ALT**	**AST**	**LDH**	**CK**	**ALT**	**AST**	**LDH**	**CK**
Before	25%	12.05	12.90	157.00	54.50	13.70	13.00	151.00	52.00
	Median	16.80	17.05	175.50	81.00	18.40	16.50	179.00	72.00
	75%	22.50	23.10	200.50	120.50	23.70	21.60	202.00	101.50
After	25%	11.55	13.30	155.50	49.00	13.00	14.00	151.00	48.00
	Median	16.75	17.70	180.00	69.50	17.10	17.60	172.00	68.00
	75%	24.55	22.80	214.00	101.50	25.25	22.55	204.00	101.00
*P* value (2-tailed)		0.824	0.887	0.079	0.000	0.299	0.460	0.399	0.153

### Heterogeneity in reducing recurrent ischemic stroke among subgroups

**Fig. 2:**
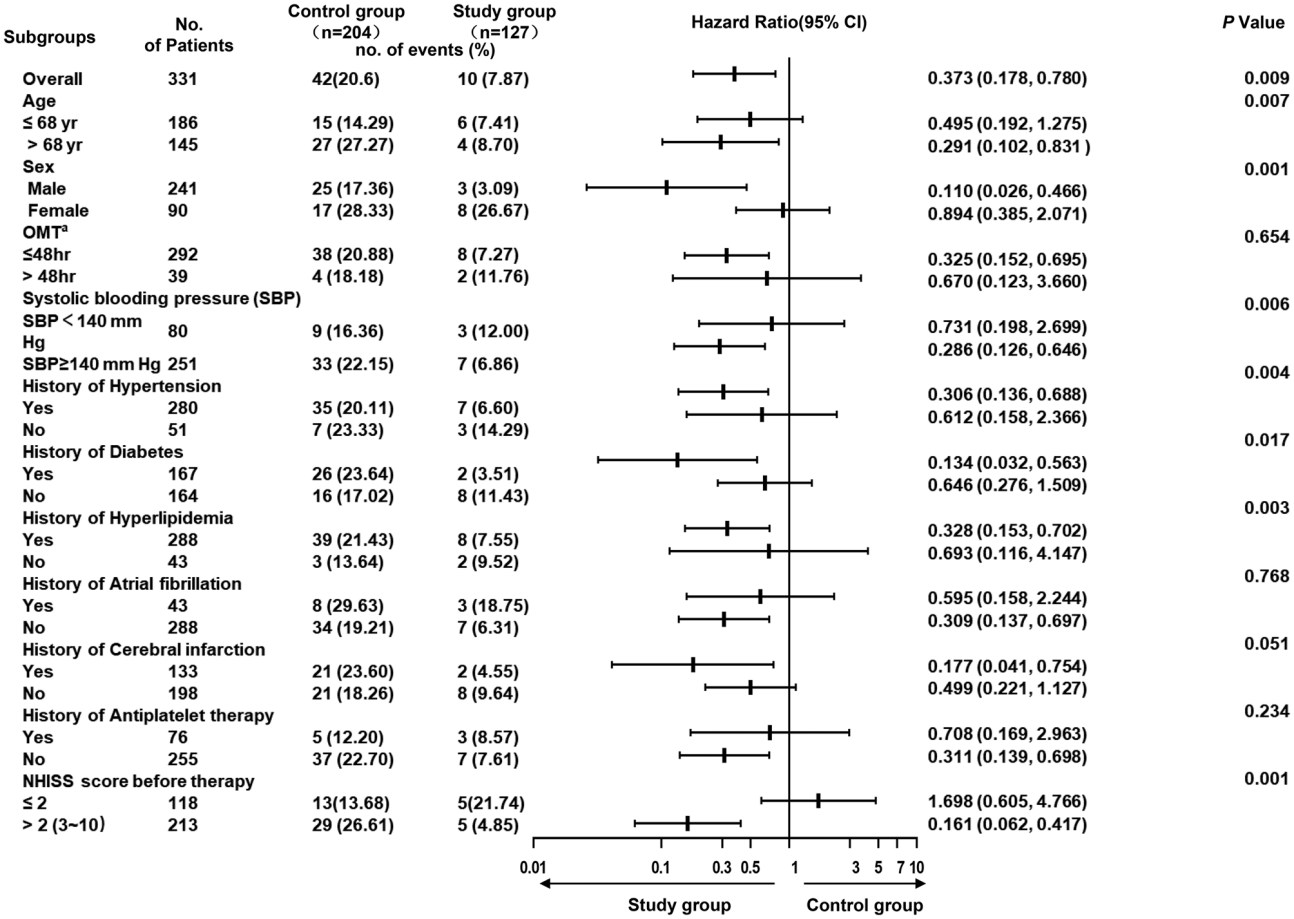
Hazard ratio for the recurrent cerebral infarction in subgroups at 90 days

To explore the heterogeneity of the two different therapy regimens in reducing the risk of recurrent ischemic stroke for AIS patients within 90 days, we performed a subgroup analysis according to the aforementioned stratification. The intensive rosuvastatin therapy plus 7-day DAPT always effectively reduced the risk of recurrent ischemic stroke, with the HR value of each subgroup located on the left side of the invalid line, except the subgroup with bNIHSS ≤ 2. It was particularly efficient in these subgroups with high-risk factors such as the elderly, hypertension, diabetes, hyperlipidemia, or prior-stroke, non-antiplatelet treatment, and high-bNIHSS (3–10 points), while it was less efficient in female, OMT > 48 h, and prior atrial fibrillation subgroups (Fig. 2).

## Discussion

In this study, compared with rosuvastatin plus SAPT, the intensive rosuvastatin therapy plus 7-day DAPT significantly reduced the risk of recurrent stroke within 90 days for patients with mild to moderate AIS, without increasing adverse events, such as bleeding, statin-induced liver injury, or SAM. The reduction was particularly significant in the subgroups with high-risk factors (the elderly [> 68 years old], hypertension, diabetes, hyperlipidemia, prior-stroke, non-antiplatelet treatment, and high bNIHSS [3–10 points]), but not significant in female, OMT > 48 h, and prior atrial fibrillation subgroups. These results should be interpreted by considering the characteristics of the therapy regimens in this study, namely, the strong inhibition of platelet aggregation and reduction of thrombosis by DAPT and the multiple effects of intensive statin therapy.

Previous study showed that statins, especially fat-soluble statins, commonly caused statin-induced liver injury and SAM, along with a three-fold or more increase in ALT, AST, or CK levels [26]. However, as a water-soluble statin, rosuvastatin only enters hepatocytes through special channel proteins on the cell membrane, rarely causing rosuvastatin-induced liver injury and SAM [27, 28]. DAPT can increase the risk of bleeding [29–31], although this is most likely to occur after 1 week of treatment [31]. Therefore, intensive rosuvastatin therapy plus 7-day DAPT significantly reduced the risk of ischemic stroke recurrence in patients with mild to moderate AIS, without increasing bleeding, statin-induced liver injury, and SAM.

In the elderly subgroup (> 68 years old), the study therapy reduced the risk of stroke recurrence more than the control therapy did, which may be related to elderly patients’ high risk of stroke [32], low self-healing ability [33], and higher dependence on effective interventions. In the male subgroup, the treatment effect of the study group was significantly better than that of the control group, while. However, there was no statistically significant difference in the female subgroup, which can be ascribed to the older age, higher prevalence of risk factors [34], higher disability severity, and worse prognosis [35–39] for female patients when the ischemic stroke occur, leading to the failure of various interventions. Moreover, the incidence rate of ischemic stroke was significantly higher in men than in women [39], which led to a relatively large sample size for the male subgroup and made it easier to obtain statistically significant results.

At the early stage of cerebral infarction, a large part of the brain tissue is still in the ischemic penumbra due to the incomplete rupture of the lipid plaque and relatively mild inflammatory storm [4]. Therefore, the earlier effective intervention measures are given, the more dormant brain cells are saved, and the more obvious clinical symptoms are relieved, which could explain why the effect of the subgroup of OMT ≤ 48 h surpassed that of the subgroup with OMT > 48 h.

The NIHSS can not only quantify the symptoms and signs of focal neurological deficit [40] but also accurately reflect the volume of cerebral infarction within a certain infarct volume [41]. The lower the score, the lighter the symptoms, and the better the prognosis. Study showed that AIS patients with a bNIHSS < 3 had a good prognosis and were not affected by intravenous thrombolysis [16]. This is consistent with our results in the subgroup with bNHISS ≤ 2, independent of the therapy regimens. However, in the subgroup with bNHISS of 3–10, the study treatment was significantly more efficient than the control treatment, indicating that the intensive rosuvastatin therapy plus 7-day DAPT regimen was more beneficial to the subgroup with high bNIHSS (3–10).

Many studies have shown that AIS patients with high-risk factors [42], such as hypertension [43–45], diabetes [46, 47], hyperlipidemia [48], and prior-stroke [49], have a significantly higher risk of recurrent stroke. The study also showed that statins can reduce the incidence of serious cardiovascular and cerebrovascular events by 20–30% in patients with high-risk factors [50], which is consistent with our results. Moreover, the study treatment reduced the risk of recurrent stroke significantly more than the control treatment did in the subgroups with hypertension, diabetes, hyperlipidemia, and prior-stroke. Additionally, the study treatment did not reduce the risk of recurrent stroke any more than the control treatment did in the atrial fibrillation subgroup, which may be related to the fact that thrombus comes from the heart [51, 52], and the best treatment regimen for these patients is anticoagulation agents [53].

Aspirin and clopidogrel are the most common antiplatelet drugs. Aspirin has an irreversible inhibitory effect on platelet aggregation through acetylated platelet cyclooxygenase, while clopidogrel, as an adenosine diphosphate receptor inhibitor, inhibits platelet aggregation. For patients who took antiplatelet agents regularly and still had AIS, the possible cause was aspirin resistance [54] or clopidogrel resistance [55], which may explain that the study treatment  can only reduce the risk of recurrent stroke significantly in patients of  the subgroup of non-antiplatelet, but not in patients of the subgroup of prior-antiplatelet.

## Limitations

This study was a single-center study with small sample size and incomplete randomized controlled design. These characteristics inevitably led to some weaknesses in the research results, which need to be confirmed by future large sample size and multi-center clinical studies.

## Conclusion

Compared with rosuvastatin plus SAPT, the intensive rosuvastatin therapy plus 7-day DAPT with aspirin and clopidogrel significantly reduced the risk of recurrent ischemic stroke within 90 days for patients with mild to moderate AIS, without increasing bleeding, statin-induced liver injury, or SAM. These effects were particularly significant in the subgroups with high-risk factors such as elderly patients (> 68 years old), patients with hypertension, diabetes, hyperlipidemia, prior-stroke, non-antiplatelet treatment, and high bNHISS scores (3–10).

## Supplementary Information

Below is the link to the electronic supplementary material.

Supplementary file1 (DOCX 11 KB)

## Data Availability

All the required data about the study are present in the manuscript. We are happy to provide additional data if the reviewer or the editor requires further data.
