# Supplementary material for: Evaluation and subgroup analysis of the efficacy and safety of intensive rosuvastatin therapy combined with dual antiplatelet therapy in patients with acute ischemic stroke
Source: Eur J Clin Pharmacol. 2022 Dec 29;79(3):389–97. doi: 10.1007/s00228-022-03442-8 (PMC9941271; doi:10.1007/s00228-022-03442-8)
Supplement: Supplementary file 1 — Supplementary file1 (DOCX 11 KB) [file 228_2022_3442_MOESM1_ESM.docx]

**Informed Consent**

Double antiplatelet therapy (DAPT) with aspirin and clopigogrel is the first choice for patients with acute ischemic stroke (AIS) when intravenous thrombolysis and arterial thrombolysis are not suitable.

Based on it, this study shortened the duration of DAPT to 7 days, and combined with intensive rosuvastatin, and compared of the effectiveness and safety of the two regimens of 21 days DAPT and intensive rosuvastatin + 7 days DAPT.

DAPT has the risk of bleeding, including cerebral hemorrhage and gastric mucosal hemorrhage, which is related to the dose and duration of DAPT; Intensive statins may cause muscle soreness, increase of muscle enzyme and liver enzyme, which is related to the characteristics (water-soluble or fat soluble), dosage and course of treatment of statins.

The patients themselves and their legal proxies were informed of the study and signed a consent form.

**Agree to the 21 day DAPT regimen**: **Relationship with Patient**：

**Agree to the 7-day DAPT + Intensive Rosuvastatin**: **Relationship with Patient:**

**Rejects the above two regimens:** **Relationship with Patient:**

**Signed on:**
